# Supplementary material for: A HER2-Displaying Virus-Like Particle Vaccine Protects from Challenge with Mammary Carcinoma Cells in a Mouse Model
Source: Vaccines (Basel). 2019 May 20;7(2):41. doi: 10.3390/vaccines7020041 (PMC6631560; doi:10.3390/vaccines7020041)
Supplement: Supplementary file 1 [file vaccines-07-00041-s001.zip › vaccines-489305 SI figures/Figure S1.pdf]

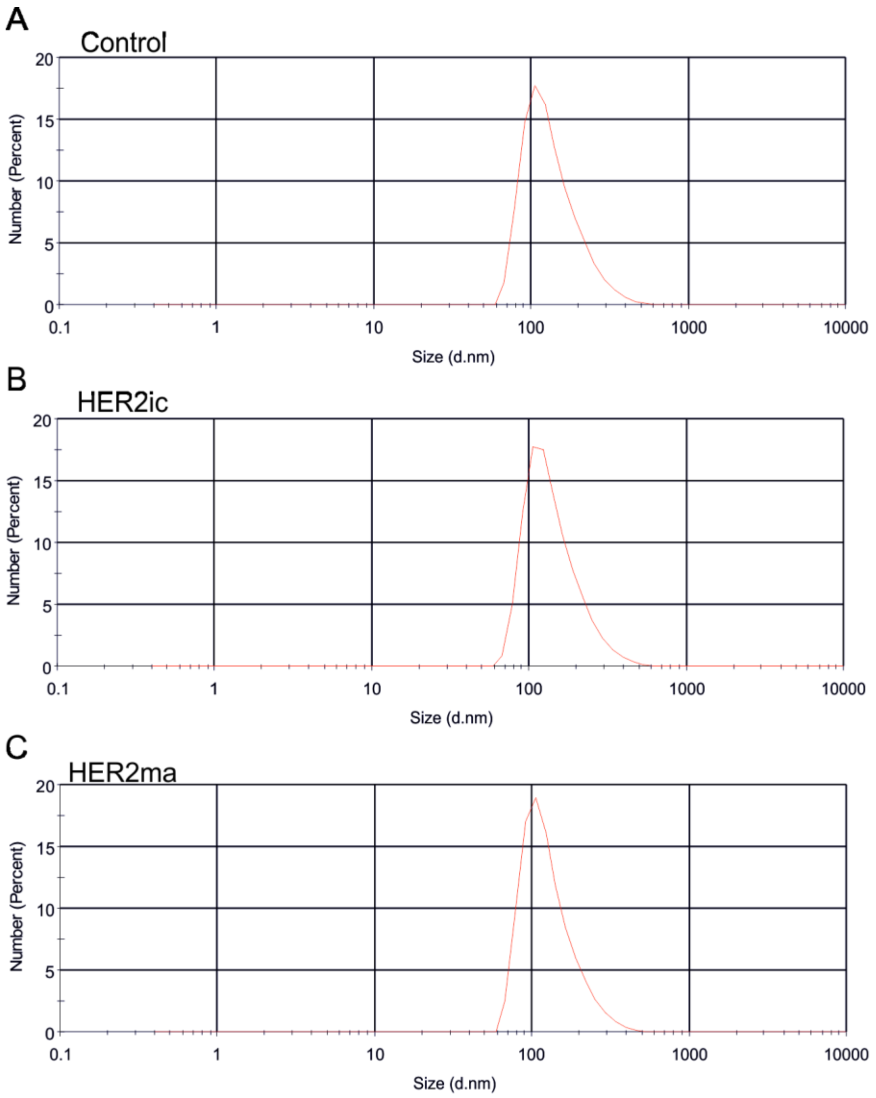

Supplementary Figure 1: DLS curves elicited by (A) Control VLPs, (B) HER2ic VLPs and (C) HER2ma VLPs. The mean diameter and homogeneity of the VLP preparations was determined by DLS using a Zetasizer (Malvern). All particles had a mean diameter size of 140 ± 10 nm and showed homogeneous distribution.
