# Supplementary material for: A HER2-Displaying Virus-Like Particle Vaccine Protects from Challenge with Mammary Carcinoma Cells in a Mouse Model
Source: Vaccines (Basel). 2019 May 20;7(2):41. doi: 10.3390/vaccines7020041 (PMC6631560; doi:10.3390/vaccines7020041)
Supplement: Supplementary file 1 [file vaccines-07-00041-s001.zip › vaccines-489305 SI figures/Figure S2.pdf]

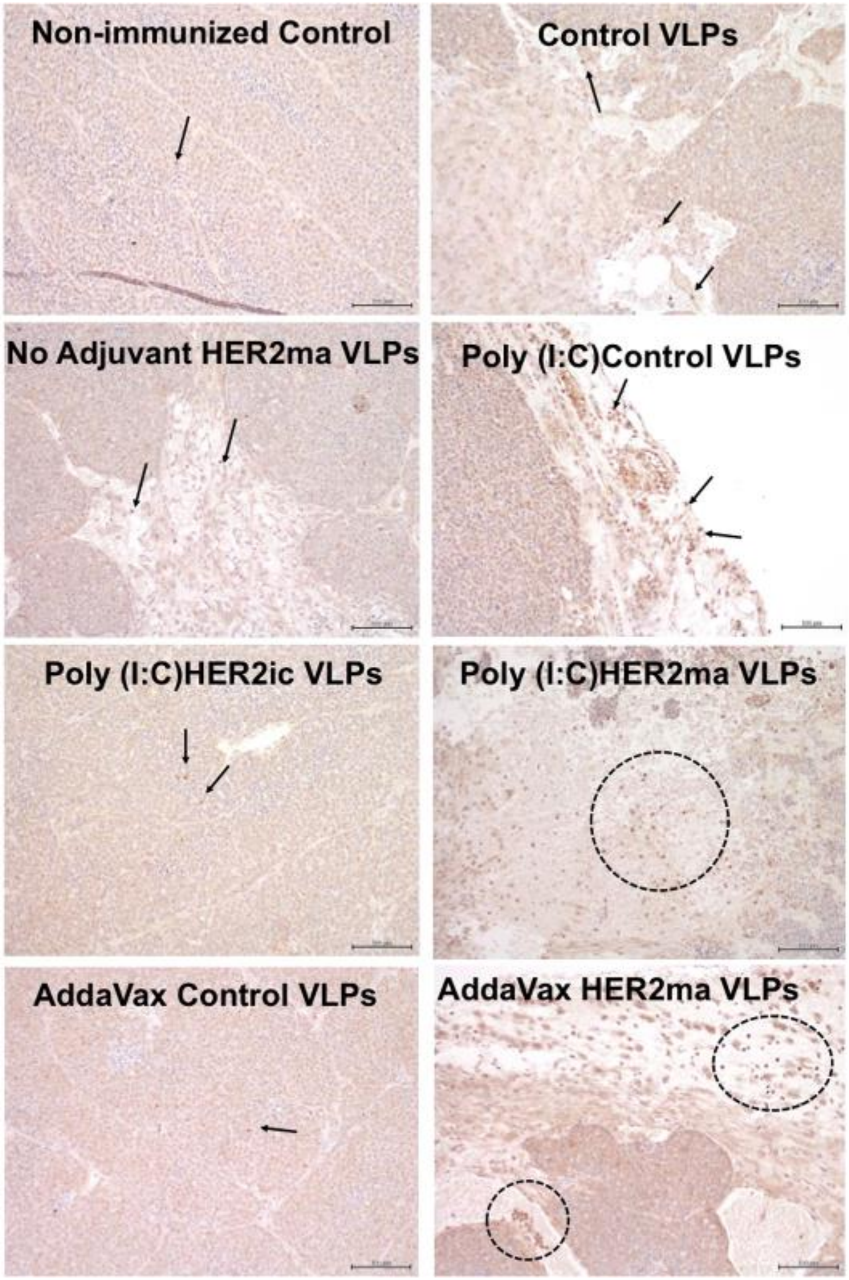

Supplementary Figure 2. Presence and distribution of tumor infiltrating CD8+ T-lymphocytes. Immunohistochemistry revealing the presence and distribution of $CD8^+$ T- lymphocytes in 5 µm-thick tumor sections. Black arrows note individual CD8+ T-cells. Rounded lines define areas with abundance of CD8+ T-cells and myeloid cells. Magnification 200X; scale bar 100 µm. **Non-immunized Control**: single CD8+ cells infiltrating neoplastic cords. It is adjacent to an area of necrosis. **Control VLPs:** intratumoral vessel with intravascular CD8+ lymphocyte (arrow). A single CD8+ lymphocyte is infiltrating the edge of a neoplastic cord. **No adjuvant HER2ma VLPs:** intratumoral stroma with stromal and intravascular CD8+ lymphocytes. **Poly (I:C) Control VLPs:** stroma around the tumor with stromal and intravascular CD8+ lymphocytes. **Poly (I:C) HER2ic VLPs:** few CD8+ lymphocytes adjacent to necrosis. **Poly (I:C) HER2ma VLPs:** intratumoral necrosis and hemorrhage with CD8+ lymphocytes. **AddaVax Control VLPs:** tumor with infiltrating CD8+ lymphocyte adjacent to necrotic area. **AddaVax HER2ma VLPs:** stroma around the tumor with moderate numbers of stromal and intravascular CD8+ lymphocytes.
