# Supplementary material for: A HER2-Displaying Virus-Like Particle Vaccine Protects from Challenge with Mammary Carcinoma Cells in a Mouse Model
Source: Vaccines (Basel). 2019 May 20;7(2):41. doi: 10.3390/vaccines7020041 (PMC6631560; doi:10.3390/vaccines7020041)
Supplement: Supplementary file 1 [file vaccines-07-00041-s001.zip › vaccines-489305 SI figures/Figure S3.pdf]

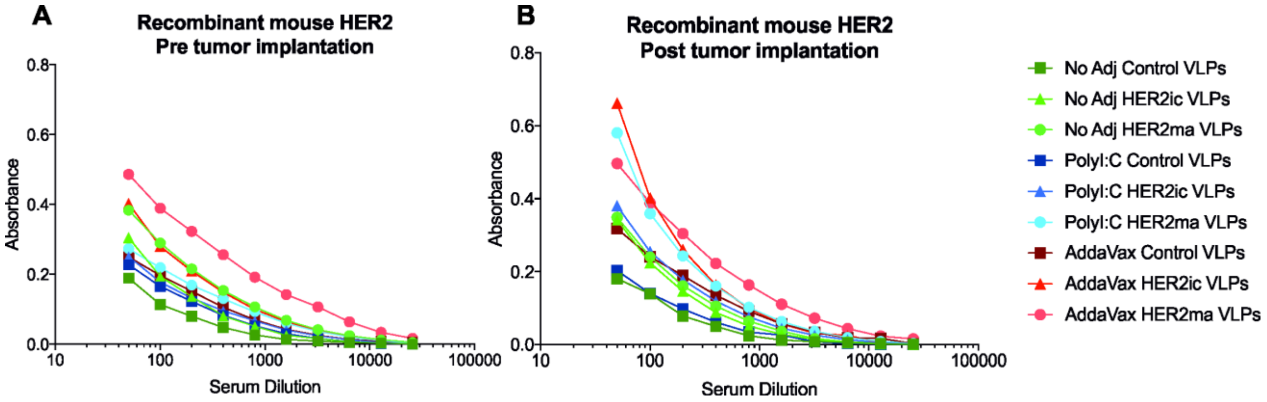

Supplementary Figure 3: Binding of serum antibodies to recombinant mouse HER2. Mice were immunized with Control, HER2ic or HER2ma VLPs in a prime-boost regimen, non-adjuvanted or in combination with Poly (I:C) or AddaVax. Serum antibodies of vaccinated mice (**A**) pre and (**B**) post tumor implantation were investigated in an ELISA against recombinant mouse HER2 protein (expressed in High Five insect cells). Results show binding of serum antibodies to recombinant mouse HER2, proofing the cross-reactivity of antibodies induced by VLP vaccinations.
