# Supplementary material for: A HER2-Displaying Virus-Like Particle Vaccine Protects from Challenge with Mammary Carcinoma Cells in a Mouse Model
Source: Vaccines (Basel). 2019 May 20;7(2):41. doi: 10.3390/vaccines7020041 (PMC6631560; doi:10.3390/vaccines7020041)
Supplement: Supplementary file 1 [file vaccines-07-00041-s001.zip › vaccines-489305 SI figures/Figure S4.pdf]

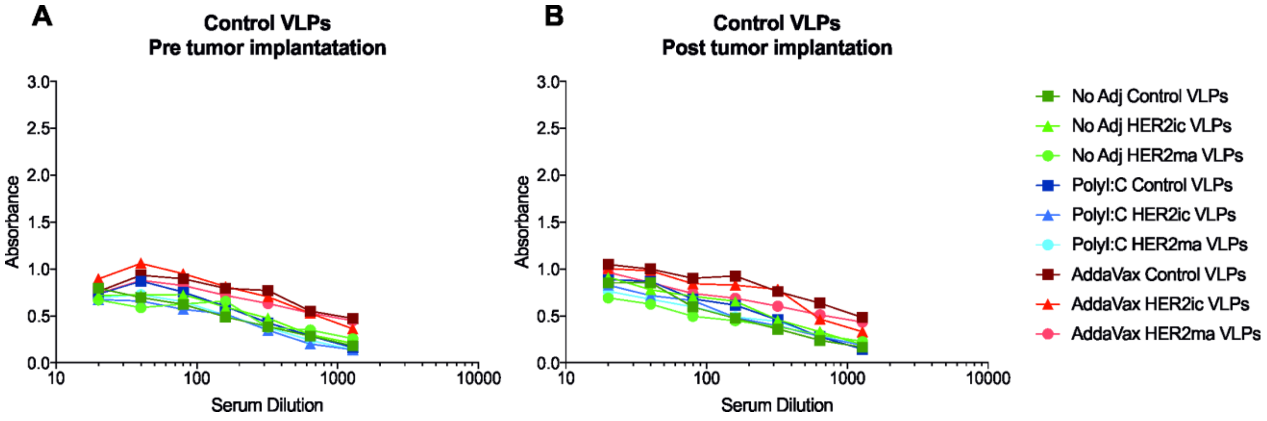

Supplementary Figure 4: Binding of serum antibodies to the carrier protein antigen (Control VLPs). Mice were immunized with Control, HER2ic or HER2ma VLPs in a prime-boost regimen, non-adjuvanted or in combination with Poly (I:C) or AddaVax. Serum antibodies of vaccinated mice (**A**) pre and (**B**) post tumor implantation were investigated in an ELISA against Control VLPs. Results show comparable low antibody titers with all immunizations, indicating that the immune response is specifically directed toward the HER2 target epitope as opposed to the carrier protein antigen.
