# Supplementary material for: A HER2-Displaying Virus-Like Particle Vaccine Protects from Challenge with Mammary Carcinoma Cells in a Mouse Model
Source: Vaccines (Basel). 2019 May 20;7(2):41. doi: 10.3390/vaccines7020041 (PMC6631560; doi:10.3390/vaccines7020041)
Supplement: Supplementary file 1 [file vaccines-07-00041-s001.zip › vaccines-489305 SI figures/Figure S5.pdf]

	Pre tumor implantation					
	No Adjuvant		Poly (I:C)		AddaVax	
	HER2ic	HER2ma	HER2ic	HER2ma	HER2ic	HER2ma
**IgG1**	0.362	0.233	1.034	0.299	1.821	0.514
**IgG2a**	2.398	2.346	2.587	1.995	2.895	2.898
**IgG2b**	0.955	0.977	0.905	0.837	2.486	1.214

	Post tumor implantation					
	No Adjuvant		Poly (I:C)		AddaVax	
	HER2ic	HER2ma	HER2ic	HER2ma	HER2ic	HER2ma
**IgG1**	0.39	0.385	1.022	0.646	1.966	0.558
**IgG2a**	2.896	2.247	2.786	1.739	2.973	2.901
**IgG2b**	1.529	1.299	2.192	1.204	2.019	0.631

Supplementary Figure 5: Raw data of anti-HER2 IgG subtype analysis. Mice were immunized with Control, HER2ic or HER2ma VLPs in a prime-boost regimen, non-adjuvanted or in combination with Poly (I:C) or AddaVax. IgG subtypes elicited by HER2ic and HER2ma VLP vaccination were analyzed in an ELISA against recombinant human HER2 using anti-IgG1, IgG2a and IgG2b secondary antibodies for detection. Absorbance values of IgG1, IgG2a and IgG2b subtypes of induced anti-HER2 antibodies elicited by indicated vaccinations are shown.
